# Hydrolyzed Bound Phenolics from Rice Bran Alleviate Hyperlipidemia and Improve Gut Microbiota Dysbiosis in High-Fat-Diet Fed Mice

**DOI:** 10.3390/nu14061277

**Published:** 2022-03-17

**Authors:** Guanghe Zhao, Ruifen Zhang, Fei Huang, Lihong Dong, Lei Liu, Xuchao Jia, Jianwei Chi, Yongxuan Ma, Mei Deng, Yanxia Chen, Qin Ma, Mingwei Zhang

**Affiliations:** 1 Sericultural & Agri-Food Research Institute, Guangdong Academy of Agricultural Sciences/Key Laboratory of Functional Foods, Ministry of Agriculture and Rural Affairs/Guangdong Key Laboratory of Agricultural Products Processing, Guangzhou 510610, China; greatriver2007@163.com (G.Z.); hf1311@163.com (F.H.); dolify@163.com (L.D.); liulei309@tom.com (L.L.); jiaxuchao@gdaas.cn (X.J.); jianweichi@126.com (J.C.); ma_yongxuan@163.com (Y.M.); monaelegant@foxmail.com (M.D.); 18016690595@163.com (Y.C.); qinma_gaas@163.com (Q.M.); 2 Life Sciences of College, Guangxi Normal University, Guilin 541006, China

**Keywords:** rice bran, hydrolyzed bound phenolics, lipid metabolism, gut microbiota

## Abstract

It has been confirmed the lipid-lowering effect of rice bran free phenolics, but it is unknown whether rice bran bound phenolics, the phenolic profile of which differs from the free ones, have a similar effect. Thus, the hypolipidemic effect and potential mechanism of hydrolyzed bound phenolics (HBP) from rice bran was investigated in this study. The results showed that HBP supplementation significantly improved serum lipid profiles of high-fat-diet fed mice. HBP inhibited the activation of nuclear receptors liver X receptor-α (LXRα), sterol regulatory element binding protein 1c (SREBP-1c), and peroxisome proliferators-activated receptors-γ (PPARγ), and, therefore, changed the expressions of their downstream genes, including LDLR, CD36, ACC1, FAS, and DGAT2 in the liver. Moreover, HBP supplementation reversed the high-fat-diet induced gut microbiota dysbiosis. These findings suggest that HBP might alleviate the hyperlipidemia via inhibiting the hepatic *de novolipogenesis*, regulating the uptake of cholesterol and fatty acid in the liver and their absorption in the gut. The attenuation of microbiota dysbiosis might contribute to the above effects.

## 1. Introduction

Epidemiological studies and meta-analyses have proved that increased intake of dietary phenolics have various health benefits of preventing or slowing chronic metabolic disorders including chronic inflammation, cardiovascular diseases, cancer, and diabetes [1]. In addition, the well-known fresh fruits and vegetables, whole grains, such as buckwheat, brown rice, and whole wheat, are also important sources of dietary phenolics. Phenolics occur naturally both in free and bound forms. In fruits and vegetables, predominant phenolics occur in free form. Conversely, more than half phenolics in whole grains are covalently linked to cell-wall dietary fiber (DF) as non-extractable bound phenolics [2,3]. Up to now, most of available studies on the health benefits of phenolics are based on free but not bound phenolics. Our previous study showed that about 25% of bound phenolics in rice bran dietary fiber could be released by gut microbes, indicating bound phenolics have as much potential to be absorbed to exert health benefits as the free ones [4]. Considering the high ration of the bound to the total phenolics, the exploration of physiological activity of bound phenolics is of a great help in understanding the contribution of phenolics to the health benefits of whole grains.

However, bound phenolics naturally coexist with DF. In order to obtain the bound phenolics separated from DF, an alkaline hydrolysis method is usually used. The extracts from the hydrolysate of bound phenolics after alkaline hydrolysis, here, are called hydrolyzed bound phenolics (HBP). The bioactivity of HBP from different materials has been investigated in a few studies. HBP, but not free phenolics, from jujube pulp exhibited pro-apoptotic effects on HepG2 cells [5]. HBP from inner shell of foxtail millet bran (BPIS) exhibited higher antimicrobial activity than the millet bran free phenolics [6]. It was also demonstrated that BPIS exhibited anti-inflammatory effects in lipopolysaccharides-induced human colon cancer HT-29 cells via promoting reactive oxygen species (ROS) accumulation and upregulating miR-149 expression to block the phosphorylation of Akt and the subsequent activation of nuclear factor-kappaB [7]. BPIS also showed antiproliferative activity towards tumor cells and worked as a chemosensitizer of 5-fluorouracil (5-Fu) towards multidrug-resistant HCT-8/5-Fu cell lines [8,9,10]. Therefore, not only free phenolics, but also HBP from many materials would be good bioactive components with potential health benefits.

As the representative of whole grains, brown rice is rich in bound phenolics distributing mainly in the bran fraction. Brown rice-contained diets were found to have hypocholesterolemic activity on rats [11]. However, the active components responsible for this effect were not clarified. Our previous study showed that free phenolics of rice bran had hypolipidemic activity in high-fat-diet fed mice [12]. HBP of rice bran showed higher antioxidant activity by cellular antioxidant assay (CAA) than free phenolics of rice bran [13]. The phenolic profiles of free and bound phenolics from rice bran were significantly different. Much higher percentages of ferulic acid and *p*-coumaric acid were detected in the bound fraction [13,14]. Ferulic acid could effectively ameliorate insulin resistance, type 2 diabetes (T2D), oxidative stress and alleviate hyperlipidemia induced by high fat diet [15,16,17]. Therefore, it is plausible that the HBP from rice bran might contribute to the lipid-lowering effects of brown rice. In the present study, the hypolipidemic effects of HBP from rice bran were investigated using high-fat-diet fed mice and the possible mechanism was also explored to give further revelation on the health benefits of whole grain brown rice.

## 2. Materials and Methods

### 2.1. Main Chemical and Reagents

Triacylglycerols (TG), Total cholesterol (TCH), Low-density lipoprotein cholesterol (LDL-C), High-density lipoprotein cholesterol (HDL-C), free fatty acids (FFA) and total bile acid (TBA) kits were purchased from Nanjing Jiancheng Bioengineering Institute (Nanjing, China). The antibodies against liver X receptor-α (LXRα) (ab176323), peroxisome proliferator activated receptor γ (PPARγ) (ab45036), sterol regulatory element binding protein 1c (SREBP-1c) (ab28481), cluster of differentiation 36 (CD36) (ab133625), acyl-CoA carboxylase 1 (ACC1) (ab72046), fatty acid synthase (FAS) (ab15285), low-density lipoprotein receptor (LDLR) (ab30532), diacylglycerol acyltransferase 2 (DGAT2) (ab237613) and Goat Anti-Rabbit IgG H&L (HRP) (ab205718) were purchased from Abcam company(Cambridge, UK).

### 2.2. Preparation of HBP from Rice Bran

HBP was prepared according to the following method. Briefly, rice bran was soaked in 80% ethanol at room temperature for 24 h. Then, the supernatant was separated and the extraction procedure was repeated two times to remove the free phenolics. The residue was mixed with 2 M NaOH and continuously shaken under a nitrogen atmosphere at room temperature for 1 h. After centrifugation (6800× *g*, 10 min), the supernate was neutralized with concentrated hydrochloric acid and extracted 5 times with ethyl acetate. The pooled ethyl acetate fractions were evaporated at 45 °C and the residue was freeze-dried to obtain the HBP. The total content of bound phenolics in HBP was 79.32 ± 0.23 g/100 g. Ferulic and *p*-coumaric acids were the predominant compounds, accounting for 65.03% and 13.05% of HBP, respectively. Vanillic, *p*-hydroxybenzonic and syringic acids together with the methyl esters of caffeic acid and ferulic acid were also detected with a total percentage of 0.24%. The remaining components in HBP were water (9.50 ± 0.51%), protein (2.21 ± 0.08%), sugar (3.44 ± 0.12%), and ash (4.65 ± 0.42%). The contents of monomer phenolics in HBP could be seen in Supplementary Table S1.

### 2.3. Animal Experiment

SPF grade 8-week-old C57BL/6J male mice were purchased from Beijing Vital River Laboratory Animal Technology Co., Ltd. (License number: SCXK (Beijing, China) 2016-0011). The mice were housed in a constant temperature of 23 ± 2 °C and humidity-controlled room with a 12 h light/dark cycle. Mice were given free access to water and food throughout the experiment. The study was conducted in accordance with the Declaration of Helsinki, and the experimental protocol were approved by the Animal Care and Use Committee, Guangdong Pharmaceutical University (Project identification code 00166980/20.3.2017). After 7 d acclimatization, mice were randomly divided into 3 groups according to body weight, with 20 animals per group. Mice in the normal control diet group (NC group) were fed with normal feed (D12450J, Research Diets, Inc., New Brunswick, NJ, USA), while those in the high-fat diet group (HF group) and high-fat diet plus HBP group (HBP group) were fed with high-fat feed (D12451, Research Diets, Inc., New Brunswick, NJ, USA) during the 14 week experimental period. HBP group mice were administered with HBP (100 mg/kg/day) dissolved in distilled water at 1.5 mg/mL by gavage every day, and distilled water was given as vehicle in the NC and HF groups.

Feces were collected during the last 3 days before mice were sacrificed and lyophilized for determining the contents of TG, TCH, and TBA. After 14 weeks’ treatment, the mice were fasted overnight and euthanized to collect blood for serum separation. The liver was excised, rinsed, weighed, and then cut into some parts. One part was fixed in 4% paraformaldehyde overnight for subsequent paraffin embedding and hematoxylin and eosin (H&E) staining. The other parts were snap-frozen in liquid nitrogen, then stored at −80 °C for real-time PCR or Western blot analysis. The epididymal fat was excised and fixed in 4% paraformaldehyde solution for H&E staining. The small intestine was cut longitudinally and the mucosa was scraped off for real-time PCR or Western blot analysis. Feces in the colon were collected and immediately stored at −80 °C for 16S rRNA sequence analysis.

### 2.4. Histological Observation of the Liver and Epididymal Fat

The histopathology of liver and epididymal fat was assessed via H&E staining following a standard experimental procedure. Briefly, tissue samples were embedded in paraffin, sectioned serially at 4 μm thickness. Afterwards, paraffin sections were deparaffinized in xylene, rehydrated in alcohol gradients, and subsequently stained with H&E. The stained sections were observed with a light microscope (Leica DMI 4000B, Heidelberger, Baden-Württemberg, Germany).

### 2.5. Serumand Feces Biochemical Assays

The levels of TG, TCH, HDL-C, LDL-C, and free fatty acids (FFA) in serum and the levels of TG, TCH, and TBA in feces were determined using their corresponding kits (Nanjing Jiancheng Bioengineering Institute).

### 2.6. Western Blot Analysis

Nuclear protein extract and cytoplasmic protein extract were obtained by homogenizing the liver tissue in ice-cold extraction buffer for nuclear protein and cytoplasmic protein, respectively, using the respective commercial kits (Beyotime Biotechnology, Shanghai, China). The protein concentrations were determined using a BCA assay Kit (Beyotime Biotechnology, Shanghai, China). Western blot analysis was conducted as the following describes. Briefly, after mixing with equal volume of 2×SDS-PAGE sample loading buffer (Beyotime Biotechnology Company, Shanghai, China), the protein extracts were heated at 95 °C for 5 min to denature the proteins. Protein samples (50 μg) were separated by SDS-PAGE and transferred to PVDF membranes. The membranes were incubated in Blocking Buffer on a shaker for 1 h at 25 °C, and then blotted with the corresponding first antibodies at 4 °C overnight. After incubation with the second antibody, the bands were visualized by enhanced chemiluminescence reagents (Millipore Corporation, Burlington, MA, USA), and subjected to the autoradiography film. Densitometry was determined with the Gel-ProAnalyzer software (Media Cybernetics, Rockville, MD, USA). Histone and β-actin were detected as the internal controls for the nuclear protein and cytoplasmic protein extracts, respectively.

### 2.7. Real Time PCR Analysis

Total RNA was isolated from the liver and small intestine mucosa samples using Trizolreagent (Aidlab, Beijing, China) and converted to cDNA by a First-Strand cDNA Reverse Transcription kit (Thermo FisherScientific, Waltham, MA, USA). The cDNA was used for quantitative real-time PCR performed on a Biosystems ViiA^TM^ 7 Real-Time PCR System (ThermoFisher Scientific, Waltham, MA, USA). The primer sequences of the genes were designed according to the sequence information from GenBank database (Table 1). The relative mRNA abundance was calculated by the 2^−ΔΔCT^ method and the values for each sample were normalized to glyceraldehyde 3-phosphate dehydrogenase (GAPDH).

### 2.8. Microbiota Analysis by 16S rRNA Gene Sequencing

Total bacteria DNA isolation, sequencing, and sequence analysis were conducted according to the method reported by Zhang, Dong et al. (2020) [18]. Briefly, the DNA was extracted from feces by the QIAamp DNA Stool Mini Kit (QIAGEN, Hilden, Germany) according to the manufacturer’s instructions. The integrity of the extracted DNA was assessed by electrophoresis in 1.5% (wt%/vol%) agarose gels. The selected samples were used as the template to amplify the V3-V4 hypervariable region of the 16S rRNA gene. Then, the amplification products were subjected to Illumina MiSeq sequencing, followed by sequence analysis of feces microbiota using QIIME (Version 2.0, Shanghai Majorbio Bio-pharm Technology Co., Ltd., Shanghai, China).

### 2.9. Statistical Analysis

All data were expressed as mean ±SD. IBM SPSS Statistic Version 20.0 (SPSS Inc., Chicago, IL, USA) was used for statistic alanalysis. Statistical comparisons were evaluated with a one-way ANOVA followed by Duncan post hoc test. Statistical results *p* < 0.05 were considered statistically significant.

## 3. Results

### 3.1. Effects of HBP on the Serum and Feces Lipid Profiles of High-Fat-Diet Fed Mice

As shown in Table 2, high-fat feeding resulted in significant increases in serum TG, TCH, LDL-C, and FFA levels of the mice, compared with NC group (*p* < 0.05). However, the above-mentioned alterations induced by a high-fat diet were alleviated in HBP group. Briefly, the levels of TG, TCH, LDL-C, and FFA of the mice in HBP group declined by 25.11%, 19.69%, 40.38%, and 39.61%, respectively. As for feces lipid profiles, HF group had significantly higher TG and slightly higher TBA levels than NC group. TBA level of HBP group was remarkably higher than that of HF group (*p* < 0.05), suggesting a decreased reabsorption of bile acids in the gut tract.

### 3.2. Effects of HBP on the Morphological Feasure of Liver and Visceral Adipose

**Figure 1:**
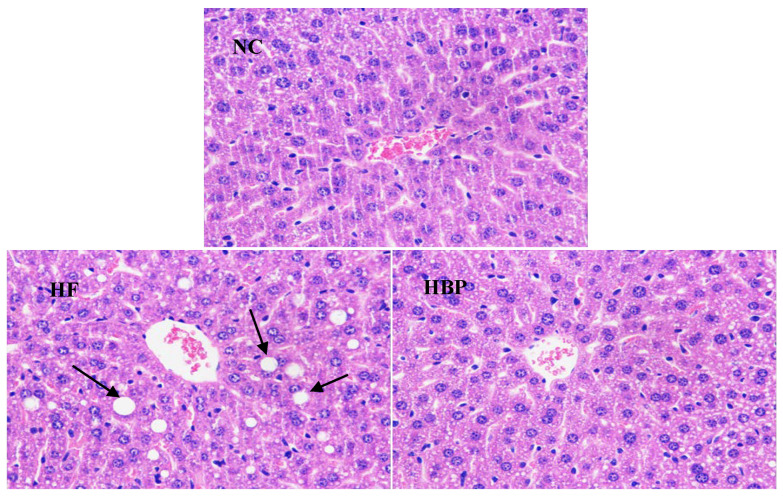
Representative microphotographs of liver tissue sections of C57BL/6J mice in NC, HF, and HBP groups (H&E staining ×200) (NC, normal control group; HF, high-fat-diet fed group; HBP, high-fat-diet fed group treated with hydrolyzed bound phenolics, the same below).

**Figure 2:**
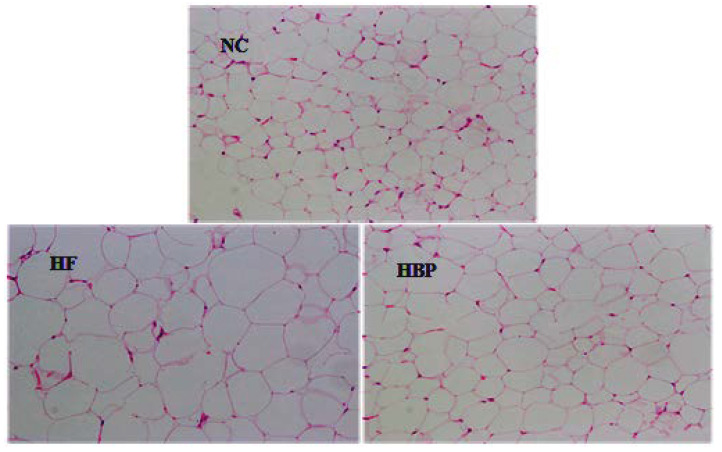
Representative photomicrographs of epididymal adipose tissue sections of C57BL/6J mice in NC, HF, and HBP groups (H&E staining ×200).

Representative microscopic morphology of hepatic tissue and epididymal adipose tissue sections subjected to H&E staining were showed in Figure 1 and Figure 2. A high-fat diet induced diffuse steatosis in the liver shown as the lipid droplets in hepatocytes in HF group, which resulted in significant cell size uniformity. Alleviated lipid accumulation was observed in HBP treated mice, exhibiting less lipid droplets in cells and orderly arrangement of cell cords in the liver lobules. The cell sizes of epididymal fat of HF group were considerably larger than those of NC group due to the accumulation of lipids. By contrast, reduced cell sizes were observed in HBP group, suggesting that HBP supplementation inhibited the lipid deposition in epididymal adipose tissue.

### 3.3. Effects of HBP on the Expression of Genes Related to Lipid Metabolism in the Liver

**Figure 3:**
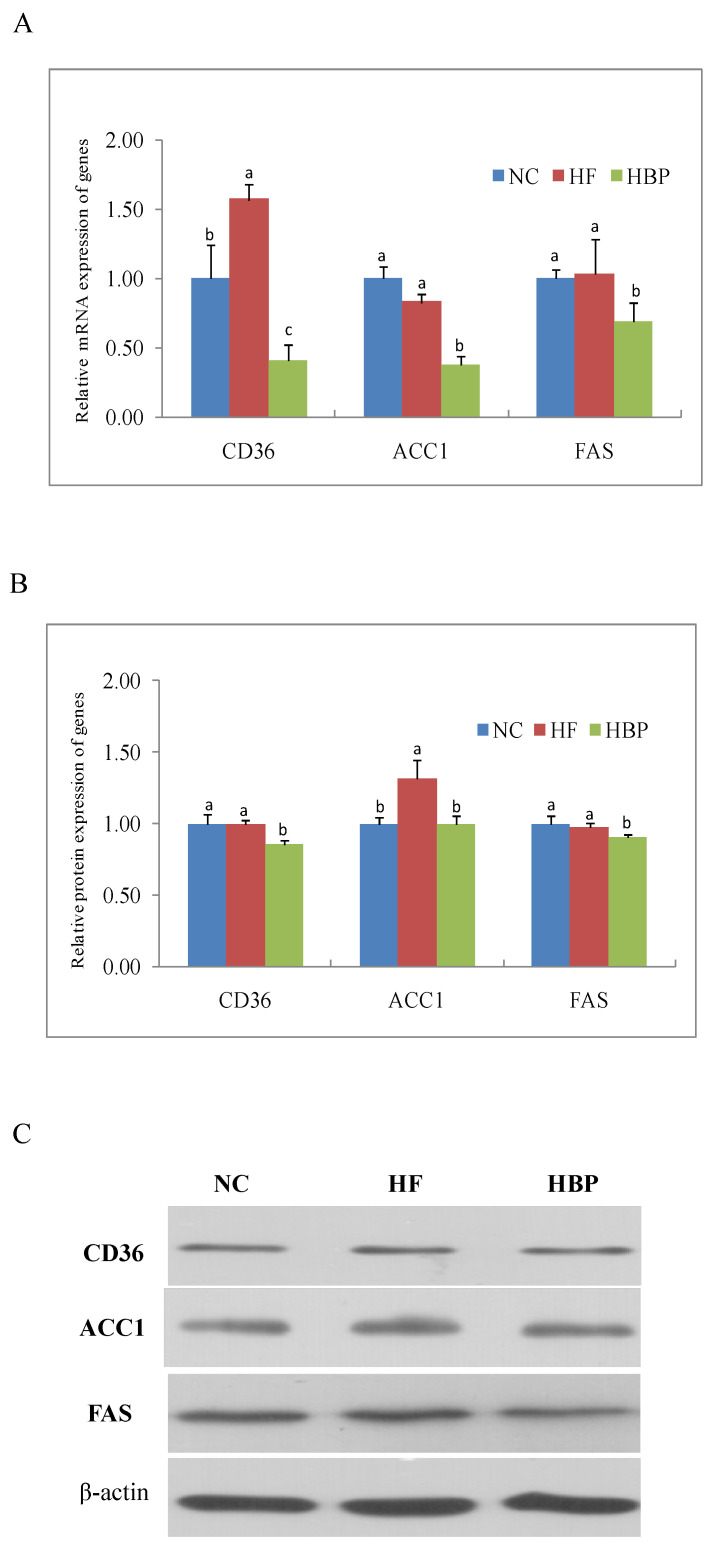
Effects of HBP on the expression of genes related with fatty acid absorption and synthesis in the liver: (**A**) Relative mRNA level of CD36, ACC1, and FAS; (**B**) relative protein levels of CD36, ACC1, and FAS; (**C**) representative bands of immunoblot. Values not sharing a letter in common are significantly different, *p* < 0.05.

**Figure 4:**
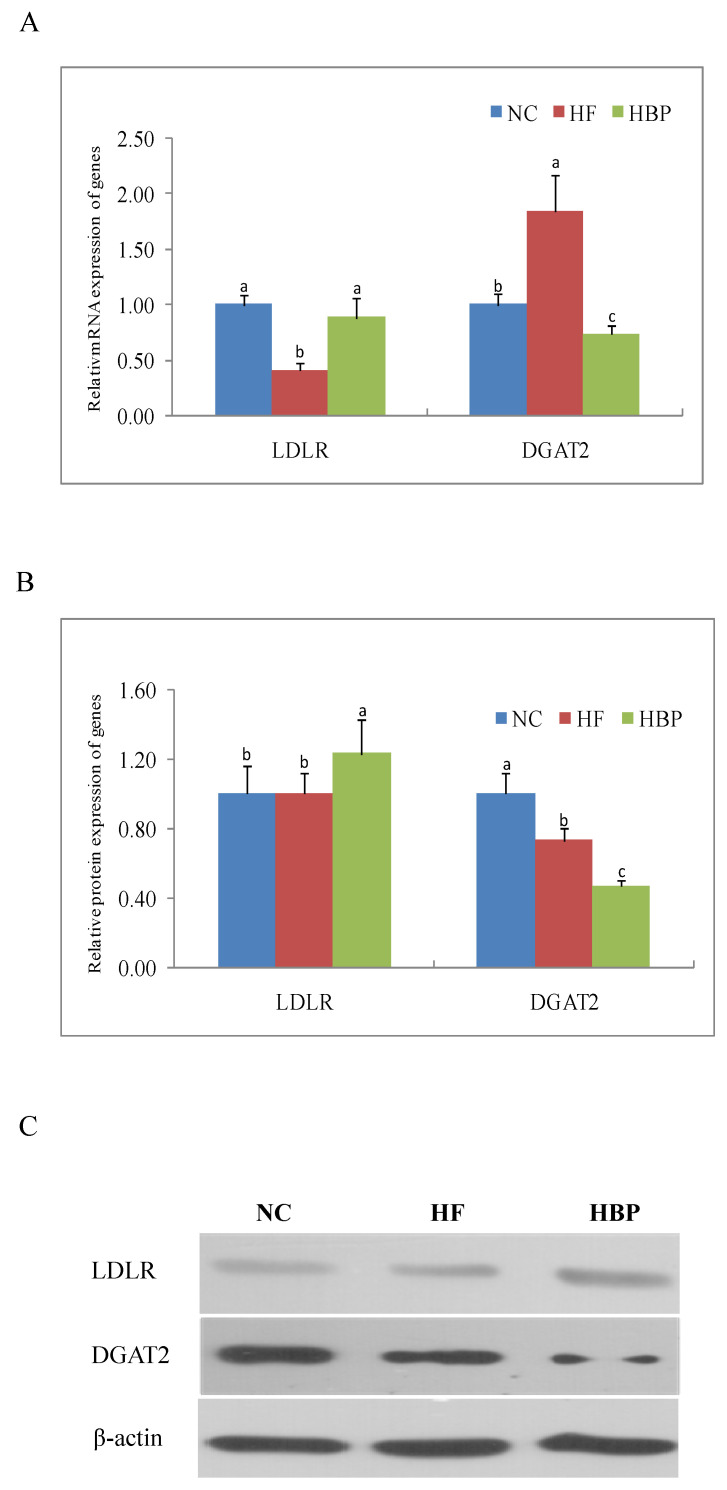
Effects of HBP on the expression of genes related with cholesterol and triglyceride metabolism in the liver: (**A**) Relative mRNA level of LDLR and DGAT2; (**B**) relative protein levels of LDLR and DGAT2; (**C**) representative bands of immunoblot. Values not sharing a letter in common are significantly different, *p* < 0.05.

The mRNA and protein levels of genes related to lipid metabolism in the liver were shown in Figure 3 and Figure 4. As shown in Figure 3, a high-fat diet significantly increased the mRNA expression of CD36 (*p* < 0.05), but had no notable effect on the mRNA expression of ACC1 and FAS (*p* > 0.05). HBP treatment remarkable decreased the mRNA expression of CD36, ACC1, and FAS in high-fat-diet fed mice (*p* < 0.05). In contrast, increased protein level of ACC1 (*p* < 0.05) but not CD36 (*p* > 0.05) was found in HF group when compared with that of NC group, which was inconsistent with the changes in mRNA of ACC1 and CD36. The FAS protein, consistent with its mRNA, showed equivalent expression levels in these two groups (*p* > 0.05). Likewise, HBP treatment inhibited the protein expressions of CD36, ACC1, and FAS in high-fat-diet fed mice (*p* < 0.05).

As shown in Figure 4, a high-fat diet significantly reduced the mRNA expression of LDLR (*p* < 0.05), but increased the mRNA expression of DGAT2 (*p* < 0.05). These changes were effectively reversed by HBP administration. HBP treatment showed similar effects on the protein expression of LDLR and DGAT2 when compared with HF group.

### 3.4. Effects of HBP on the Activation of Nuclear Receptors Related to Lipid Metabolism in the Liver

**Figure 5:**
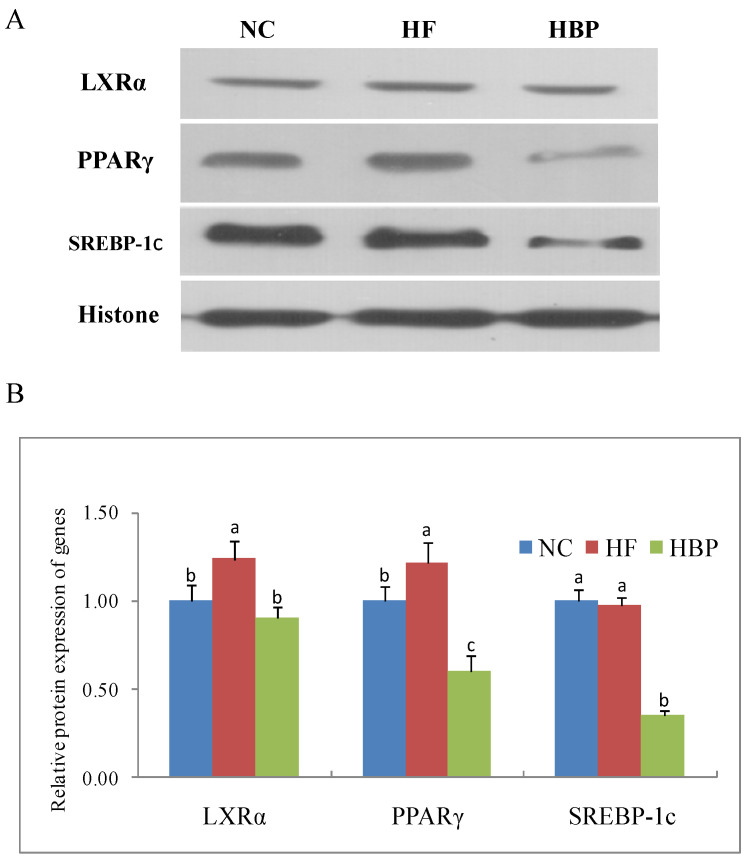
Effects of HBP on the nuclear translocation of transcriptional factors in the liver: (**A**) Representative bands of immunoblot blot; (**B**) relative protein levels of LXRα, PPARγ, and SREBP-1c in nuclear protein. Values not sharing a letter in common are significantly different, *p* < 0.05.

As shown in Figure 5, a high-fat diet significantly increased the nuclear protein levels of LXRα and PPARγ (*p* < 0.05), but had no effect on the nuclear translocation of SREBP-1c (*p* > 0.05). Compared with HF group, HBP administration repressed the nuclear translocation of the LXRα, PPARγ, and SREBP-1c (*p* < 0.05).

### 3.5. Effects of HBP on the Genes Related to Lipid Metabolism in the Gut

**Figure 6:**
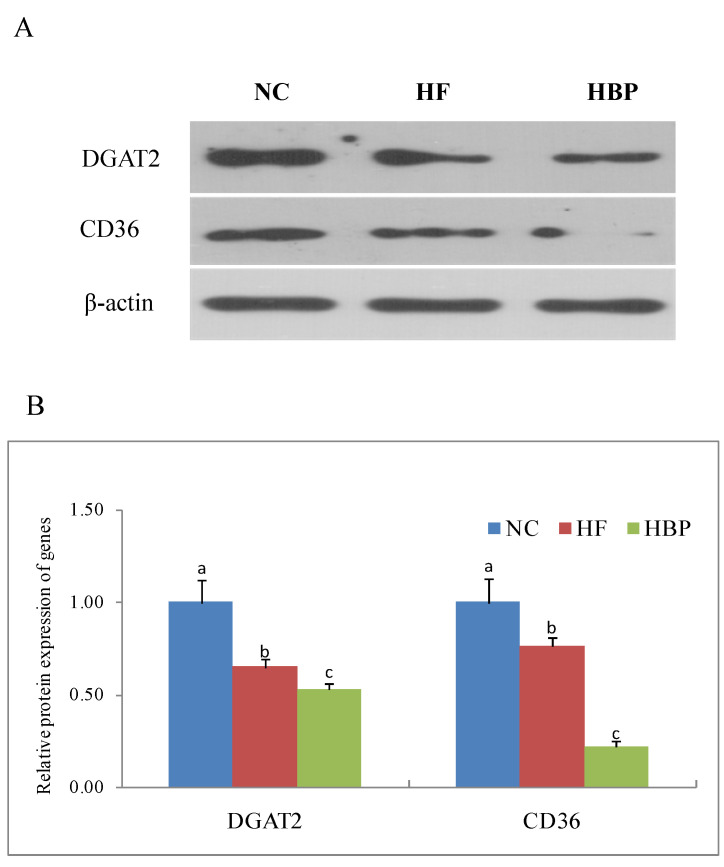
Effects of HBP on the protein expression of DGAT2 and CD36 in the intestinal mucosa of high-fat-diet fed mice: (**A**) Representative bands of immunoblot; (**B**) relative protein levels of DGAT2 and CD36. Values not sharing a letter in common are significantly different, *p* < 0.05.

As shown in Figure 6, the protein expressions of DGAT2 and CD36 in the small intestine mucosa of HF group mice were significantly lower than those of NC group (*p* < 0.05). Furtherly, decreased protein expressions were observed in HBP group (*p* < 0.05).

### 3.6. Assessment of Fecal Microbial Community Structure by 16S rRNA Sequencing

**Figure 7:**
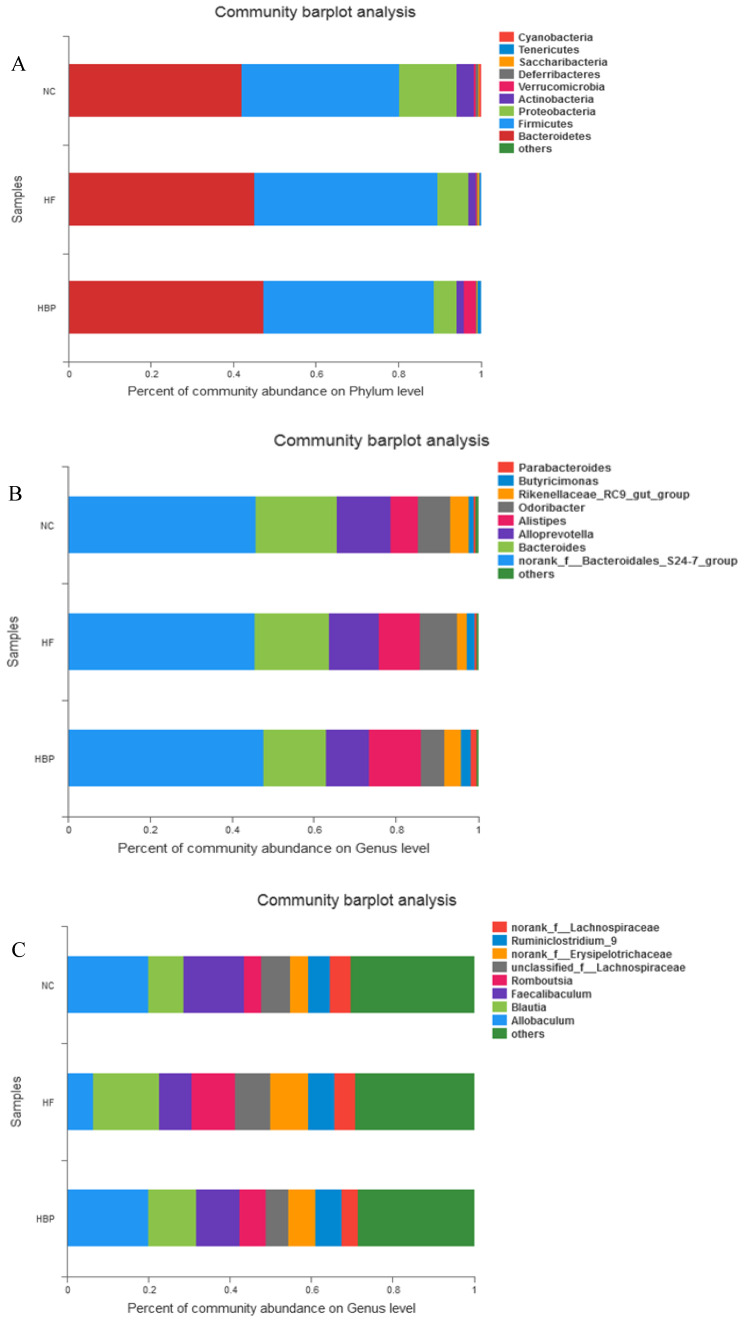
Effects of HBP on the relative abundance of bacteria at different levels in the feces of NC, HF, and HBP groups mice: (**A**) The differences of 9 main bacteria phyla of gut microbiota of mice; (**B**) the differences in the relative abundance of bacteria at genus level in the phylum *Bacteroidetes*; (**C**) the differences in the relative abundance of bacteria at genus level in the phylum *Firmicutes*.

As shown in Supplement Figure S1, the Ace index of HF group was significantly lower than those of NC group and HBP group (*p* < 0.05). These results indicated that a high-fat diet notably reduced the alpha diversity within the microbial community (*p* < 0.05), whereas the supplementation of HBP markedly increased this diversity (*p* < 0.05). Nine main bacteria phyla were identified in all fecal samples (Figure 7A). The phyla of *Bacteroidetes* and *Firmicutes* accounted for 80.37–89.66% of the reads, followed by *Proteobacteria* (5.69–13.96%). The radios of *Bacteroidetes*/*Firmicutes* were 1.10, 1.01, and 1.14 in the NC, HF, and HBP groups, respectively.

At the genus level, all 103 detected genera were shared by all fecal samples. For the *Bacteroidetes* phylum (Figure 7B), a high-fat diet decreased the relative abundance of *Bacteroides* and *Rikenellaceae_RC9_gut_group* and increased the relative abundance of *Alistipes*, *Odoribacter*, *Butyricimonas,* and *Parabacteroides*. For the *Firmicutes* phylum (Figure 7C), a high-fat diet decreased the relative abundance of *Allobaculum* and *Faecalibaculum* and increased the relative abundance of *Romboutsia*, *unclassified_f_Lachnospiraceae*, *norank_f_Erysipelotrichaceae,* and *Ruminiclostridium_9.* However, HBP supplementation effectively reversed these changes.

**Figure 8:**
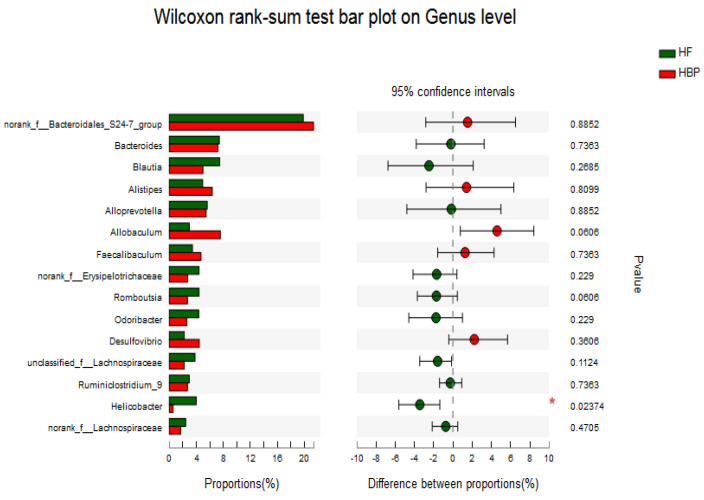
Species differences at genus of gut microbiota in the feces of HF and HBP groups mice.

To furtherly compare the difference in gut microbiota between HF and HBP groups at the genus level, Wilcoxon rank-sum test was performed. As can be seen from Figure 8, the relative abundance of *Helicobacter* in HBP group was significantly lower than that in HF group (*p* < 0.05). There was no difference in the relative abundance of other microbial genera between the two groups (*p* > 0.05).

## 4. Discussion

Our results showed that HBP supplementation effectively alleviated hyperlipidemia induced by a high-fat diet, exhibiting the improvement of blood lipid profile together with the alleviation of lipid accumulation in the liver and visceral adipose tissue. The liver is the predominant organ of *de novolipogenesis* (DNL), and plays apivotal role in regulating the lipid metabolism. Excessive uptake and synthesis of lipids in the liver will result in a breakdown of the lipid metabolism homeostasis. ACC and FAS are key enzymes responsible for the hepatic DNL. ACC catalyzes the carboxylation of acetyl coenzyme A to produce malonyl coenzyme A, and the latter is used as the substrates by FAS for the synthesis of fatty acid [19]. HBP treatment inhibited the mRNA and protein expression of ACC1 and FAS in the liver of high-fat-diet fed mice in the present study. Therefore, the reduced DNL in the liver also accounted for the hypolipidemic activity of HBP.

LDLR is a cell membrane glycoprotein that is mainly responsible for the binding and internalizing of circulating cholesterol-containing lipoprotein particles, such as VLDL and LDL, part of which are excreted from our body after conversion into bile acids, and, therefore, the hepatic level of LDLR is important for maintaining cholesterol homeostasis in mammals. The supplementation of HBP significantly elevated the mRNA and protein expression of LDLR of high-fat-diet fed mice, which was consistent with the reduced serum levels of TCH and LDL-C in this group (Table 2). The higher contents of TBA in the feces of HBP-treated mice might be attributed to the increased bioconversion of cholesterol due to the LDLR-mediated uptake of lipoprotein particles (Table 2). The scavenger receptor CD36 in hepatocytes mediates the flux of blood FFA to the liver [20]. It has previously been reported that a high-fat diet can increase the FFA level in serum and, subsequently, induce the expression of CD36 in the liver [21]. The elevations of serum FFA and mRNA expression of CD36 in mice liver were also found in the HF group in this study, which accounted for the excessive lipid accumulation in the liver. Decreased mRNA and protein expression of CD36 was found in HBP group. The results indicated that HBP administration might inhibit the excessive uptake of FFA and the subsequent lipid overaccumulation in the liver by downregulating CD36. The fatty acids deriving from the hepatic uptake and DNL could be involved in the catabolic pathway of β-oxidation or in the synthesis of TG for the subsequent assembly of VLDL, which is secreted into the blood by hepatocytes. DGAT1/2 are the acyltransferases catalyzing the re-esterification of diacylglycerol to TG. The mRNA and protein expression of DGAT2 was downregulated by the supplementation of HBP, which effectively ameliorated TG accumulation in the liver (Figure 1).

The hepatic lipid metabolism is regulated by several nuclear receptors, such as LXRs, SREBPs, and peroxisome proliferator-activated receptors (PPARs) [22,23,24]. SREBP-1c is responsible for the synthesis of fatty acids through regulating its downstream genes ACC and FAS, and for the synthesis of TG by regulating the expression of DGAT [22,25]. Additionally, the expression of CD36 is also modulated by PPARγ and liver—specifically by LXRα [26,27]. Therefore, the nuclear translocation of nuclear factors LXRα, PPARγ, and SREBP-1c were investigated in the present study. Our results showed that HBP treatment consistently inhibited the nuclear translocation of these nuclear factors in the liver when compared to the HF group mice. Interestingly, hepatic LXRα was not influenced by the intake of free phenolic extracts from rice bran (FPE) in our previous study [12]. The inconsistent effects of HBP and FPE from rice bran on hepatic LXRα might be attributed to their different phenolic profiles. HBP might inhibit the SREBP-1c expression and consequently downregulate the mRNA and protein expression of ACC1 and FAS in the liver. We also reported that FPE reduced the expression SREBP-1c in high-fat-diet fed mice. It has been shown that the expression of SREBP-1c is regulated by LXRα. The synchronized downregulation of SREBP-1c and LXRα in HBP group in the present study indicated that HBP treatment could inhibit the hepatic DNL in the high-fat-diet fed mice through the LXRα/SREBP-1c signaling pathway.

The gut is the most important location for the absorption of dietary fats, which is partly regulated by gut DGAT2 and CD36. Besides catalyzing the DNL in the liver, DGAT2 promotes the conversion of diet fats into chylomicrons in the gut tract [28]. CD36 is highly expressed on the brush border membrane of the proximal intestine villi [29,30], facilitating fatty acid uptake and directing fatty acids for chylomicron production [31]. The downregulation of gut DGAT2 and CD36 in HBP group suggested that HBP could suppress the absorption of dietary fats in gut tract of mice fed with a high-fat diet.

Intestinal microflora has been proved to play an important role in host health by providing energy and nutrition and by strengthening mucosalimmunity. In this study, high-throughput sequencing technology was used to study the possible effects of HBP treatment on microbial communities. The results showed that a high-fat diet markedly reduced the alpha diversity of gut microbiota of mice, which was effectively reversed by the administration of HBP. Our findings were consistent with the previous reports [32,33]. *Bacteroides* and *Firmicutes* are the most abundant phyla in the gut bacteria. It was speculated that the decreased ratio of *Bacteroides*/*Firmicutes* was observed in high-fat-diet fed mice and found to be associated with obesity [34,35]. HBP-treated mice showed increased *Bacteroides*/*Firmicutes* ratio, indicating HBP supplementation might alleviate the intestinal microbial dysbiosis.

High-fat diet consumption caused the abundance changes in some bacterial genera closely-related with glucose and lipid metabolism in the gut tract of mice. The increased abundance of *Rikenellaceae RC9* might be related with the lipid metabolism homeostasis [36], while the increase in *Erysipelotrichaceae* abundance indicated the imbalance of lipid metabolism in the host to some extent [37,38]. *Allobaculum* could improve insulin sensitivity, increase the level of gut short-chain fatty acids (SCFA) and reduce systemic inflammation, and consequently prevent the occurrence of metabolic diseases [39]. Additionally, *Blautialuti* (B8) abundance has been reported to be positively correlated with the levels of serum LDL-C and TCH [40]. The alteration of gut microflora of the HBP-treated mice in the above-mentioned genera was consistent with the hypolipidemic effect of HBP. However, it could not be figured out that the alteration in gut microflora was a cause or result of the improved blood lipid profiles. The causal relationship between gut microflora regulation and lipid metabolism homeostasis resulting from HBP administration remains to be investigated in the future study.

Reportedly, *Parabacteroides, Bacteroides, Romboutsia* are closely related with gut diseases [41,42,43]. The adverse abundance changes in these genera induced by a high-fat diet were effectively reversed by HBP treatment, suggesting HBP supplementation might improve gut diseases of mice fed with a high-fat diet.

## 5. Conclusions

In summary, the present study demonstrated that HBP from rice bran could inhibit the hepatic DNL and the uptake of cholesterol and fatty acid in the high-fat-diet fed mice through the LXRα/SREBP-1c signaling pathway. Further, HBP treatment suppressed the absorption of dietary fats in the gut by downregulating the gene expressions of DGAT2 and CD36. On the whole, the possible hypolipidemic mechanism of HBP could be summarized as Supplement Figure S2. Moreover, HBP supplementation stimulated the growth of *Rikenellaceae RC9* and *Allobaculum*, inhibited the growth of *Erysipelotrichaceae* and *Blautialuti* (B8) and increased the *Bacteroides*/*Firmicutes* ratio. Therefore, the bound phenolics in whole grain brown rice and its products might play key role in maintaining lipid metabolism homeostasis.

## Figures and Tables

**Table 1:** Primer sequences and the length of the target genes.

Target Genes	Primer Sequences (5′–3′)		Product Length
GAPDH	Forward	AGGAGCGAGACCCCACTAACA	247 bp
	Reverse	AGGGGGGCTAAGCAGTTGGT	
CD36	Forward	CCCAGATGACGTGGCAAAGA	159 bp
	Reverse	GAAGGCTCAAAGATGGCTCCA	
ACC1	Forward	GCTAAACCAGCACTCCCGATTC	279 bp
	Reverse	GCTGGAGAAGCCACAGTGAAATC	
FAS	Forward	AGGAGGTGGTGATAGCCGGTA	298 bp
	Reverse	CGGAGTGAGGCTGGGTTGATA	
LDLR	Forward	AGACTCATGCAGCAGGAACGA	271 bp
	Reverse	GAAGTCATCCTGGGAGCACGT	
DGAT2	Forward	TAAAGGATCTGCCCTGTCACG	163 bp
	Reverse	CAGGAAGGATAGGACCCATTGTA	

**Table 2:** Effects of a 14-week treatment with hydrolyzed bound phenolics from rice bran on serum and fecal lipid profiles of C57BL/6J mice fed a high-fat diet.

	NC	HF	HBP
Serum			
TG (mmol/L)	1.68 ± 0.12 b	2.31 ± 0.19 a	1.73 ± 0.19 b
TCH (mmol/L)	3.72 ± 0.14 c	5.13 ± 0.33 a	4.12 ± 0.18 b
LDL-C (mmol/L)	2.01 ± 0.20 b	2.60 ± 0.22 a	1.55 ± 0.14 c
HDL-C (mmol/L)	1.45 ± 0.14	1.44 ± 0.16	1.38 ± 0.07
FFA (mmol/L)	1.08 ± 0.08 b	1.54 ± 0.12 a	0.93 ± 0.07 c
Feces			
TG (mg/g dry feces)	4.34 ± 0.24 b	5.15 ± 0.12 a	4.70 ± 0.35 ab
TCH (mg/g dry feces)	2.31 ± 0.26	2.36 ± 0.23	2.29 ± 0.18
TBA (μmol/g dry feces)	26.45 ± 0.85 b	28.36 ± 1.76 b	39.33 ± 1.82 a

NC, normal diet; HF, high-fat diet; HBP, high-fat diet plus hydrolyzed bound phenolics. The data are presented as the mean ± SD (*n* = 20). Data with different letters in the same line are statistically different (*p* < 0.05). TG, triglyceride; TCH, total cholesterol; LDL-C, low-density lipoprotein-cholesterol; HDL-C, high-density lipoprotein-cholesterol; FFA, free fatty acid; TBA, total bile acid.

## Data Availability

The data presented in this study cannot be shared at this time as the data also forms part of an ongoing study.

## Supplementary Materials

The following supporting information can be downloaded at: https://www.mdpi.com/article/10.3390/nu14061277/s1, Table S1: The contents of monomer phenolics in hydrolyzed bound phenolics (HBP) from rice bran. Figure S1: The alpha diversity of gut microbiota of mice from different groups. (NC, normal control group; HF, high-fat-diet fed group; HBP, high-fat-diet fed group treated with hydrolyzed bound phenolics). Figure S2: The possible hypolipidemic mechanism of HBP from rice bran.

## Abbreviations

ACC, acyl-CoA carboxylase; ALT, alanine aminotransferase; AST, aspartate transaminase; CD36, cluster of differentiation 36; DGAT2, diacylglycerol O-acyltransferase 2; FAS, fatty acid synthase; FFA, free fatty acids; HBP, hydrolyzed bound phenolics; LDL-C, low-density lipoprotein cholesterol; LXRα, liver X receptors-α; LDLR, low-density lipoprotein receptor; SREBP-1c, sterol regulatory element binding protein 1c; TCH, total cholesterol; TG, triacylglycerols; TBA, total bile acid.
