# Supplementary material for: Hydrolyzed Bound Phenolics from Rice Bran Alleviate Hyperlipidemia and Improve Gut Microbiota Dysbiosis in High-Fat-Diet Fed Mice
Source: Nutrients. 2022 Mar 17;14(6):1277. doi: 10.3390/nu14061277 (PMC8953714; doi:10.3390/nu14061277)
Supplement: Supplementary file 1 [file nutrients-14-01277-s001.zip › nutrients-1564547-supplementary.pdf]

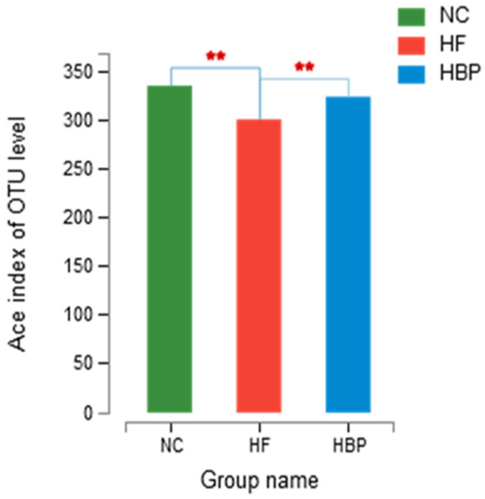

Figure S1. The alpha diversity of gut microbiota of mice from different groups. (NC, normal control group; HF, high-fat-diet fed group; HBP, high-fat-diet fed group treated with hydrolyzed bound phenolics).

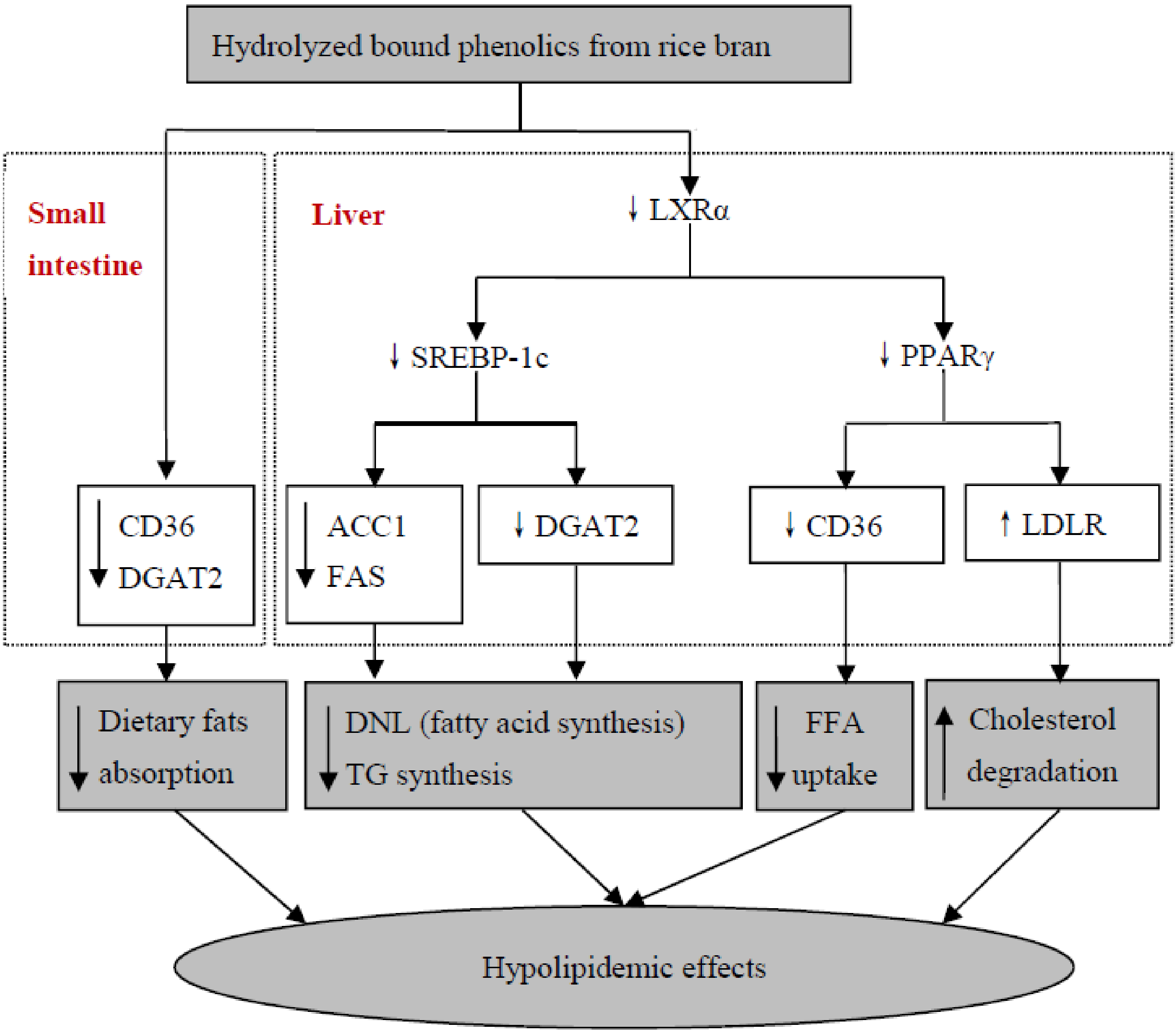

Figure S2. The possible hypolipidemic mechanism of HBP from rice bran.

Table S1. The contents of monomer phenolics in hydrolyzed bound phenolics. (HBP) from rice bran.

Varieties	Contents (g/100g)
p-Hydroxybenzonic acid	0.04 ± 0.00
Vanillic acid	0.14 ± 0.00
Syringic acid	0.06 ± 0.00
p-Coumaric acid	13.95 ± 0.07
Ferulic acid	65.03 ± 0.88
Caffeic acid methyl	0.07 ± 0.00
Ferulic acid methyl ester	0.03 ± 0.00
